# Computer modeling of whole-cell voltage-clamp analyses to delineate guidelines for good practice of manual and automated patch-clamp

**DOI:** 10.1038/s41598-021-82077-8

**Published:** 2021-02-08

**Authors:** Jérôme Montnach, Maxime Lorenzini, Adrien Lesage, Isabelle Simon, Sébastien Nicolas, Eléonore Moreau, Céline Marionneau, Isabelle Baró, Michel De Waard, Gildas Loussouarn

**Affiliations:** 1 Université de Nantes, CNRS, INSERM, l’institut du thorax, F-44000 Nantes, France; 2 LabEx “Ion Channels, Science & Therapeutics”, 06560 Valbonne, France; 3 Present Address: Laboratoire Signalisation Fonctionnelle des Canaux Ioniques et des Récepteurs (SiFCIR), UPRES EA 2647, USC INRA 1330, SFR QUASAV 4207, UFR Sciences, Université d’Angers, Angers, France

**Keywords:** Cardiovascular biology, Biophysics, Computational biophysics, Permeation and transport

## Abstract

The patch-clamp technique and more recently the high throughput patch-clamp technique have contributed to major advances in the characterization of ion channels. However, the whole-cell voltage-clamp technique presents certain limits that need to be considered for robust data generation. One major caveat is that increasing current amplitude profoundly impacts the accuracy of the biophysical analyses of macroscopic ion currents under study. Using mathematical kinetic models of a cardiac voltage-gated sodium channel and a cardiac voltage-gated potassium channel, we demonstrated how large current amplitude and series resistance artefacts induce an undetected alteration in the actual membrane potential and affect the characterization of voltage-dependent activation and inactivation processes. We also computed how dose–response curves are hindered by high current amplitudes. This is of high interest since stable cell lines frequently demonstrating high current amplitudes are used for safety pharmacology using the high throughput patch-clamp technique. It is therefore critical to set experimental limits for current amplitude recordings to prevent inaccuracy in the characterization of channel properties or drug activity, such limits being different from one channel type to another. Based on the predictions generated by the kinetic models, we draw simple guidelines for good practice of whole-cell voltage-clamp recordings.

## Introduction

The patch-clamp technique has contributed to major advances in the characterization of ion channel biophysical properties and pharmacology, thanks to the versatility of the readouts: (i) unitary currents allowing the study of a single channel conductance, open probability and kinetics, and (ii) whole-cell currents allowing characterization of a population of channels, their pharmacology, the macroscopic properties of the gates, but also the gating kinetics^1,2^.

**Figure 1:**
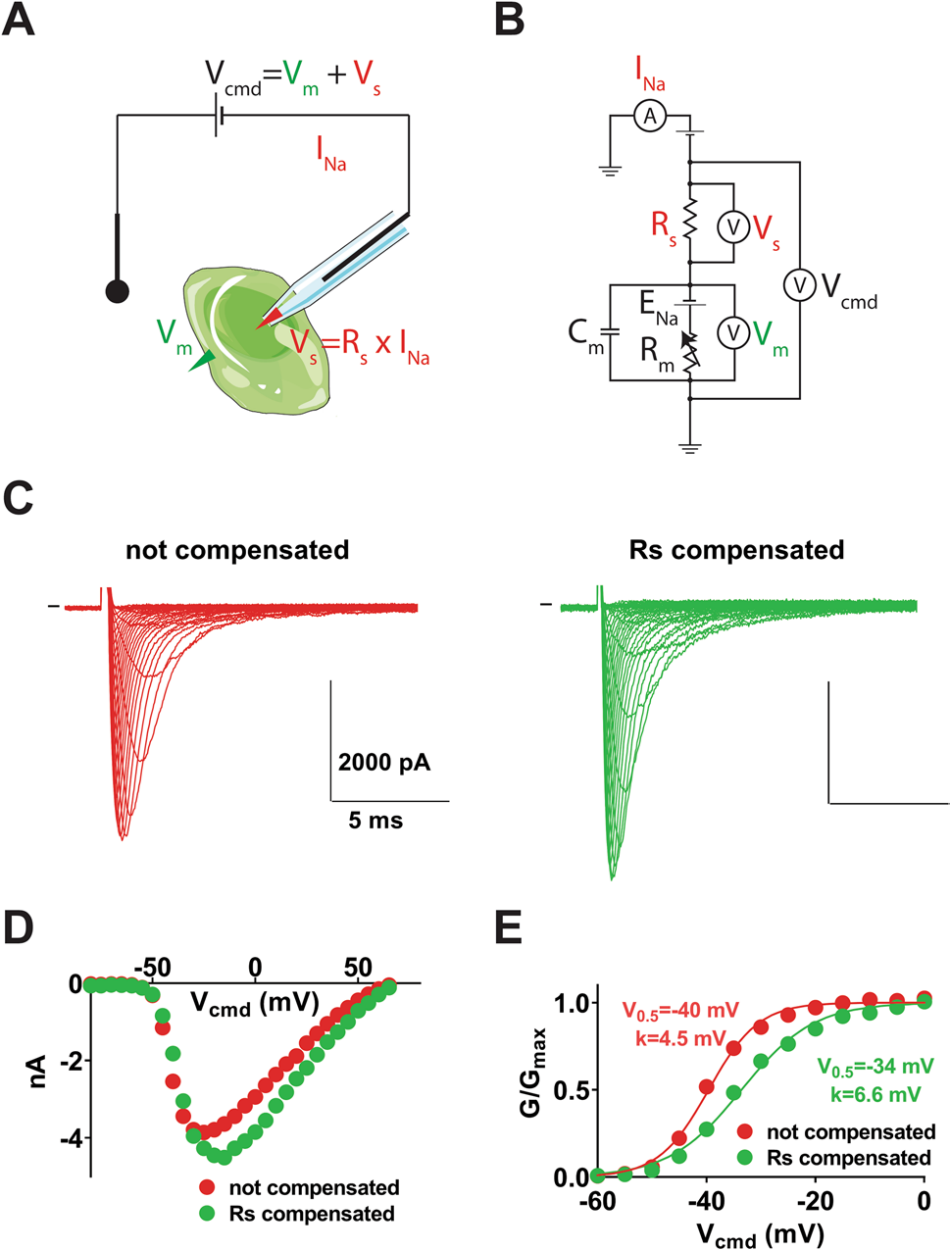
**(A)** Simplified scheme of a cell studied in voltage-clamp**,** illustrating how the potential clamped between the two electrodes is split between the membrane potential and a potential generated at the pipette tip. (**B)** Scheme of the equivalent electrical circuit. Pipette capacitance and leak current due to imperfect seal between the pipette and the cell have been omitted for the sake of clarity. R_m_: cell resistance for Na^+^. This value depends on the Na^+^ channel expression level and the fraction of expressed Na^+^ channels that are open (the higher the value, the less active are the Na^+^ channels). The fraction of channels in the open state depends on voltage and time. E_Na_: value of the reversal potential for Na^+^ (determined by intra- and extracellular Na^+^ concentrations according to the Nernst equation); Cm: cell capacitance (the higher the value, the larger is the cell). (**C**) Left, representative, superimposed recordings of heterologously-expressed Na_v_1.5 during an activation voltage protocol (from HP = − 100 mV depolarization to steps from − 80 to + 65 mV), with no compensation. Right, recordings of the same cell with R_S_ compensation. (**D**) I/V curves in the two conditions presented in **(C)**. (**E**) Activation curves in the two conditions presented in **(C)**.

As for any technique, some practical limits have to be taken into account. As schematized in Fig. 1A,B, a major caveat in the whole-cell configuration of the voltage-clamp technique is due to the fact that the pipette tip in manual patch-clamp, or the glass perforation in planar automated patch-clamp, creates a series resistance (R_S_) in the order of the MΩ. Consequently, according to the Ohm’s law, when a current flowing through the pipette is in the order of the nA, it leads to a voltage deviation of several mV at the pipette tip or the glass perforation. The actual voltage applied to the cell membrane (V_m_) is therefore different than the voltage clamped by the amplifier and applied between the two electrodes (pipette and bath electrodes, V_cmd_). This leads for example to an erroneous characterization of a channel voltage-dependent activation process.

This caveat was described early on when the patch-clamp technique was developed^3^. However, with the development of automated patch-clamp for the industry and academia, the technique that was formerly used exclusively by specialized electrophysiologists, has been popularized to scientists that are not always aware of the limits of the technique. In that respect, we now extensively witness new publications that report ionic currents in the range of several nA, that undoubtedly have led to incorrect voltage clamp and erroneous conclusions. Early on, this problem was partially solved by the development of amplifiers with the capacity to add a potential equivalent to the lost one (V_S_), a function which is called R_S_ compensation^4^. Examples of Na^+^ currents generated by Na_V_1.5 voltage-gated Na^+^ channels, recorded from a transfected COS-7 cell, with and without R_S_ compensation, are shown in Fig. 1C–E. These recordings illustrate the kind of errors that can be induced in evaluating activation in the absence of R_S_ compensation. However, compensation rarely reaches 100% and some high-throughput systems have limited compensation abilities, to avoid over-compensation and consequent current oscillation that can lead to seal disruption.

Here, we used a mathematical model to study in detail the impact of various levels of R_S_ and current amplitude on the steady-state activation and dose–response curves of the cardiac voltage-dependent Na^+^ current I_Na_, as well as the steady-state activation curve of the cardiac voltage-dependent K^+^ current I_to_. We then predicted the impact of various levels of R_S_ on the Na^+^ current activation parameters and compared this prediction to whole-cell voltage-clamp recordings obtained in manual patch-clamp analyses of cells transiently expressing Na_v_1.5 channels. Finally, we looked at the impact of R_S_ in whole-cell voltage-clamp recordings of Na_v_1.5 currents obtained in automated patch-clamp using the Nanion SyncroPatch 384PE. This study highlights potential incorrect interpretations of the data and allows proposing simple guidelines for the study of voltage-gated channels in patch-clamp, which will help in the design of experiments and in the rationalization of data analyses to avoid misinterpretations.

The aim of this study was thus to use kinetic models of specific ion currents to generate current ‘recordings’ that take into account the voltage error made using the whole-cell configuration of the patch-clamp technique. We then used and compared these data and experimental observations to propose simple rules for good quality patch-clamp recordings.

## Results

In order to calculate the current (I) recorded at a given voltage, in a cell, we used Hodgkin–Huxley models of voltage-gated channels^5^. For this calculation, we need to determine the actual membrane potential (V_m_) but we only know the potential applied between the pipette and reference electrodes (V_cmd_), illustrated in Fig. 1A,B. The voltage error between V_m_ and V_cmd_ is the voltage generated at the pipette tip (V_S_), which depends on the series resistance (R_S_) and the current.

$${V}_{m}= {V}_{cmd}- {R}_{s}\times I$$

I is the resultant of the membrane resistance variations, due to channels opening or closing, R_m_. For voltage-gated channels, R_m_ varies with voltage, V_m_, and time.

Since I is a function of V_m_, through channels voltage-dependence, and V_m_ depends on I (cf. the equation above), the value of I can only be obtained through an iterative calculation at each time step (see supplemental information with a limited number of equations, for further details).

**Figure 2:**
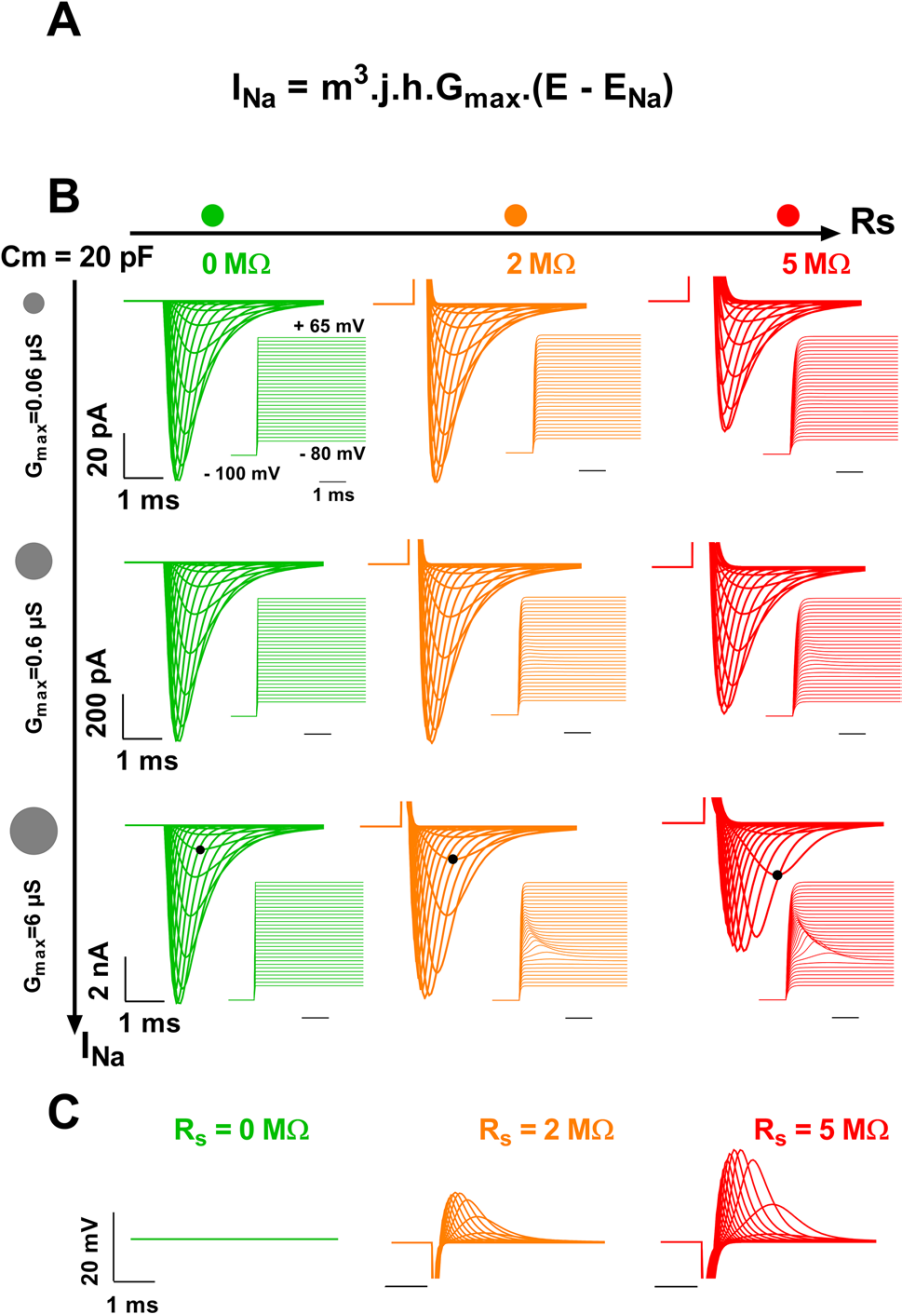
Kinetic model of cardiac I_Na_ current (Na_v_1.5)—computed effects of increasing series resistance and current amplitude range on current recordings. **(A)** Expression of the Na^+^ current depending on the activation gate (m^3^), inactivation gate (j.h), the maximal conductance (G_max_) and the reversal potential for Na^+^ (E_Na_)^5,16^. (**B)** Superimposed computed I_Na_, for increasing current amplitude range (I_Na_, see vertical scales, top to bottom) and series resistance (R_S_, left to right). The activation voltage protocol shown (in inset, holding potential: − 100 mV; 50-ms pulse at the indicated potentials; one sweep every 2 s) corresponds to the potential at the membrane, not the command potential between the two electrodes. It is thus altered when (R_S_ x I) is elevated, its maximum deflection reaching 26 mV at V_cmd_ = − 40 mV (cf text). (**C)** Superimposed computed voltage deviation for the highest amplitude (10 nA) and various Rs, as in **(B)**.

We started to model the current conducted by cardiac voltage-gated Na^+^ channels (Na_v_1.5 for the vast majority) for a combination of series resistance (R_S_) values and current amplitude ranges (depending on the amount of active channels in the membrane, Fig. 2A,B). First, when R_S_ is null (the ideal condition, which can almost be reached experimentally if R_S_ compensation is close to 100%), the voltage error is null and the shapes of the recordings are identical, independent of the current amplitude (in green in Fig. 2B). Consistent with the voltage error being proportional to both R_S_ and current amplitude values, we observed that combined increase in R_S_ and current amplitude leads to alteration in the current traces, due to a deviation of V_m_ from V_cmd_ (Fig. 2B). When R_S_ is equal to 2 MΩ (in orange), alterations in the shape of the currents are observed only when current amplitude reaches several nA (high expression of ion channels, bottom), with, for instance, time to peak at − 45 mV increasing from 0.9 ms (at R_S_ = 0 MΩ) to 1.15 ms (at R_S_ = 2 MΩ). When increasing R_S_ to 5 MΩ, alterations are minor in the medium range of current, but are emphasized when currents are large (middle and bottom, in red), with time to peak at -45 mV reaching 1.6 ms. As illustrated in Fig. 2B, when I and R_S_ are elevated, the voltage applied to the membrane, V_m_, can reach − 14 mV, whereas the applied voltage command, V_cmd_, is − 40 mV (bottom right, Vm inset). Thus, in these conditions, the voltage deviation represents 26 mV at the peak of the effect, which is not insignificant (Fig. 2C).

**Figure 3:**
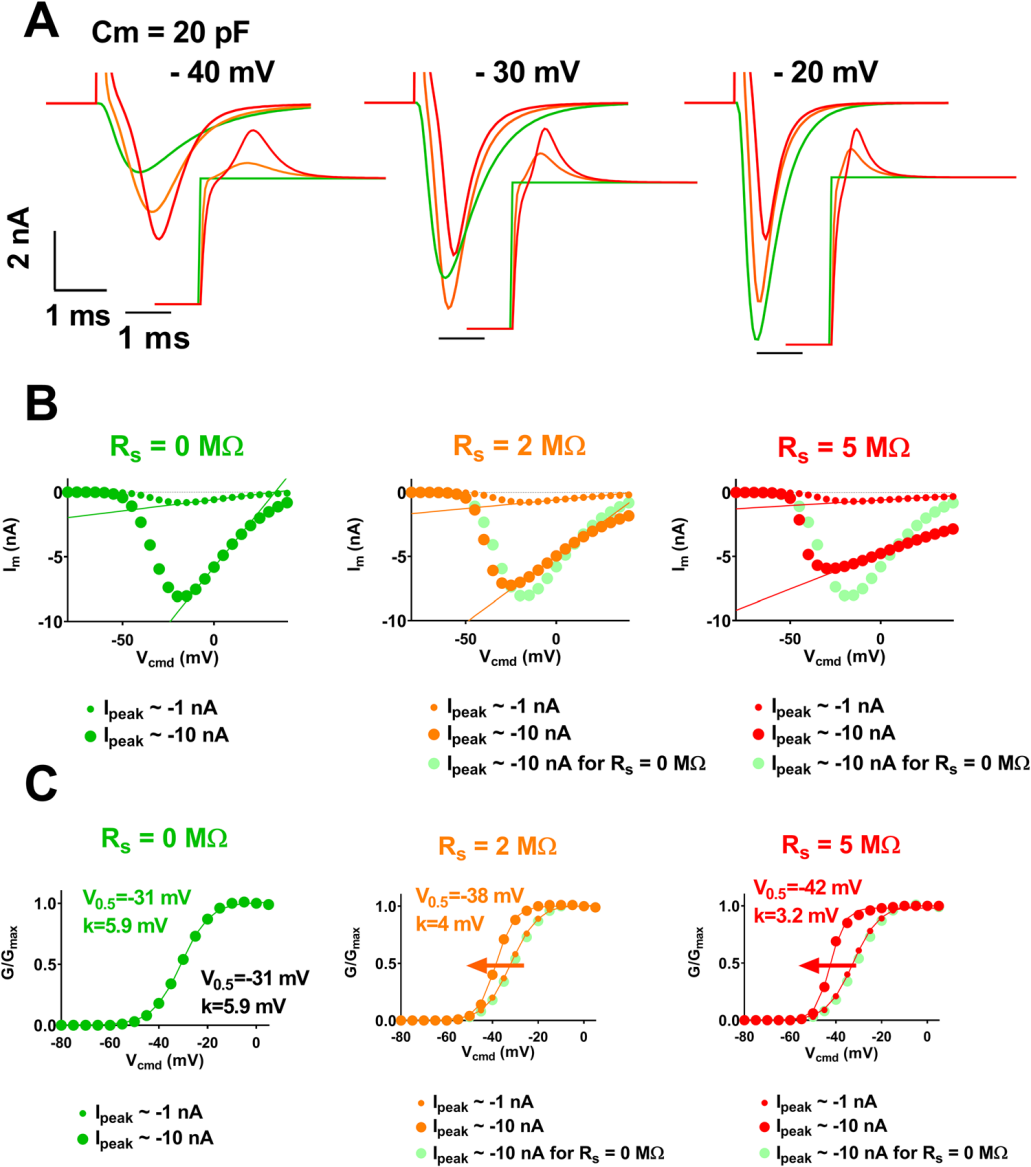
Kinetic model of cardiac I_Na_ current (Na_v_1.5)—computed effects of increasing series resistance and current amplitude range on apparent current parameters. (**A)** Computed time course of I_Na_ current at the indicated command potential, for various values of R_S_, as indicated by colors. (**B)** Peak current/voltage (I/V) relationships in the three conditions. Optimal I/V curve is repeated (light green), allowing easy comparison. Straight lines are fits of the I/V curves when voltage-dependent activation is achieved. The slope of the linear function corresponds to the maximal conductance. (**C**) Activation curves (G/G_max_ vs V_cmd_) in various conditions as indicated by color and symbol size. Theoretical half-activation potential (V_0.5_) and slope (k) values in black. V_0.5_ and k for R_S_ of 0, 2 and 5 MΩ and current range of 10 nA are indicated in colors.

The impact of R_S_ on current amplitude is the highest for large amplitudes (Fig. 3A, 10-nA range), e.g. at potentials between − 40 and − 20 mV. At such potentials, activation and inactivation time courses are clearly altered by high R_S_ values. Altogether, this leads to major artefactual modifications of the current–voltage and activation curves (Fig. 3B,C). Indeed, except when R_S_ is null, increasing the current amplitude range, from 1 to 10 nA, shifts the voltage-dependence of activation (V_0.5_) towards hyperpolarized potentials (Fig. 3C). For the largest currents, series resistance of 2 and 5 MΩ induces − 7 mV and − 11 mV shifts of V_0.5_, respectively. The slope factor k is also drastically reduced by a factor of 1.5 and 1.8, respectively.

**Figure 4:**
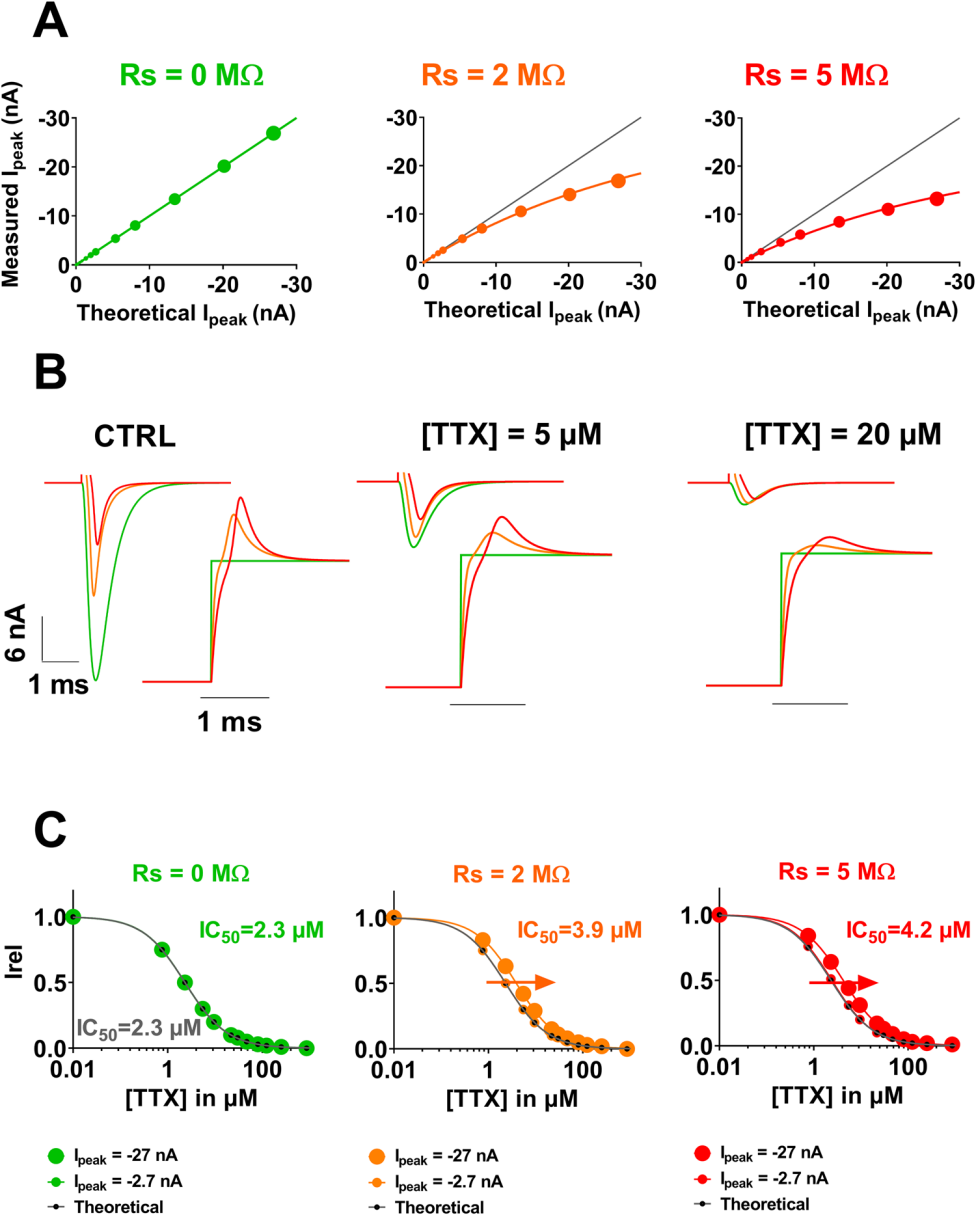
Kinetic model of cardiac I_Na_ current (Na_v_1.5)—computed effects of increasing series resistance and current amplitude on apparent TTX effects. **(A)** Relationships between the theoretical values of peak I_Na_ (with no voltage error) and the measured values of I_Na_ for three different values of R_S_ (0, 2 and 5 MΩ). (**B)** Computed recordings of the I_Na_ current at the indicated TTX concentrations, for various values of Rs, as indicated by colors. (**C**) Computed dose–response curves for three R_S_ conditions (R_S_ = 0, 2 and 5 MΩ) and two amplitude ranges (I peak =  − 2.7 and − 27 nA).

Besides impact on the characteristics of voltage-dependent activation, R_S_ may also impact channel pharmacological characteristics. In order to model this impact, we established, for various values of R_S_, the relationship between the theoretical values of the peak Na^+^ current, I_peak_ (with no voltage error) and the measured values of I_peak_. We calculated this relationship at a potential that could be used to establish the dose–response curve, here -20 mV. First, when R_S_ is null, the voltage error is null and both values (theoretical and computed values) are the same. As R_S_ increases, the measured I_Na_ curve is inflected accordingly (Fig. 4A, middle and right).

We used this relationship to look at the impact of R_S_ on the apparent effects of a channel blocker—tetrodotoxin (TTX)—on the Na^+^ current. We started with published data on TTX^6^ to generate the theoretical (R_S_ = 0 MΩ) dose–response curve and current traces in the presence of various concentrations of TTX (Fig. 4B,C, green). Then we used the relationship between the theoretical and measured values of I_peak_ at R_S_ of 2 and 5 MΩ (established in Fig. 4A), to build the dose–response curves, for these R_S_ values (see “Methods” section for details). In the absence of TTX (Fig. 4B, left), current amplitude is high and voltage-clamp is not efficient, thus there are major differences between theoretical (green) and measured (orange and red) amplitudes. When inhibitor concentration increases, remaining current amplitudes decrease and the voltage-clamp improves. Hence, with higher TTX doses the theoretical and measured values become closer, independent of the R_S_ values (Fig. 4B, right). This leads to an artefactual shift of the resulting dose–response curve towards higher concentrations (Fig. 4C). For I_peak_ = − 27 nA, R_S_ of 2 and 5 MΩ induce an increase of IC_50_ by a factor of 1.7 and 1.8, respectively. For low I_peak_ (− 2.7 nA), these modifications are minimal.

**Figure 5:**
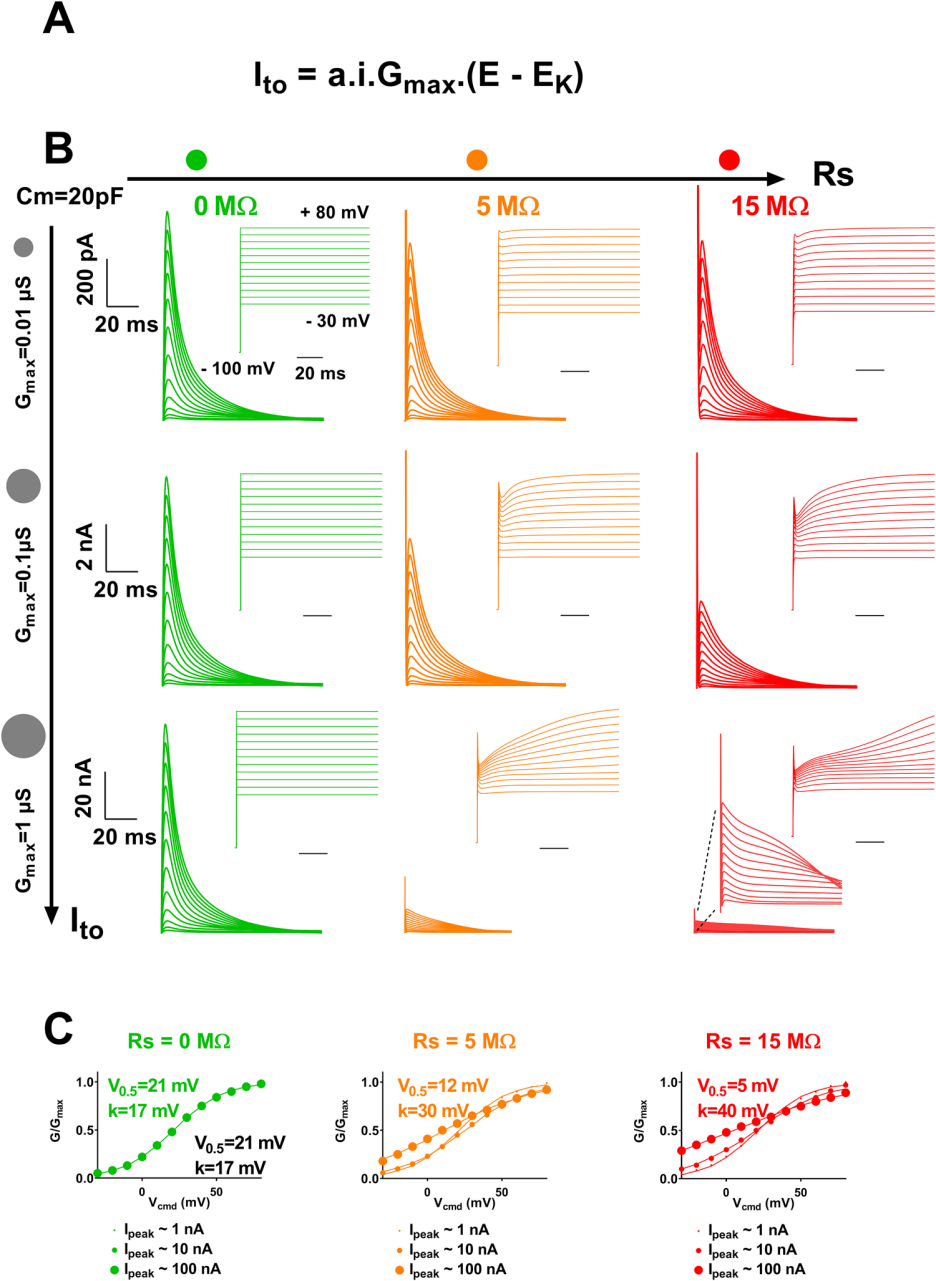
Kinetic model of cardiac I_to_ current—computed effects of increasing series resistance and current amplitude on current recordings. **(A)** Expression of the K^+^ current depending on the activation gate (a), inactivation gate (i), the maximal conductance (G_max_) and the reversal potential for K^+^ (E_K_)^5^. (**B)** Computed superimposed recordings of the I_to_ current, for various current amplitudes and series resistances (R_S_), as indicated. The activation voltage protocol shown (holding potential: − 100 mV; 100-ms pulse at the indicated potentials; one sweep every 2 s) corresponds to the potential experienced by the membrane (V_m_), not the potential between the two electrodes (V_cmd_). It is thus altered when (Rs x I) is elevated. (**C)** Activation curves (G/G_max_ vs V_cmd_) in the three conditions represented in **(B)**. Theoretical half-activation potentials (V_0.5_) and slopes (k) are indicated in black. V_0.5_ and k for R_S_ of 0, 5 and 15 MΩ and I peak of 100 nA are indicated in colors.

We applied the same modeling strategy to study the impact of R_S_ on the ‘measurement’ of the voltage-gated K^+^ current I_to_ , using a Hodgkin-Huxley model of this current^5^ (Fig. 5A). As for the Na^+^ current, we modeled the I_to_ current for a combination of values of series resistance and current amplitude. Again, when R_S_ is null, the voltage error is null and the shapes of the recordings are identical, independent of the current amplitude (in green in Fig. 5B). Consistent with voltage error being proportional to both R_S_ and current amplitude, we observed that combined increase in R_S_ and current amplitude leads to alteration in the recordings, due to a deviation of V_m_ from V_cmd_ (Fig. 5B). I_to_ current characteristics, nonetheless, are less sensitive to R_S_ and current amplitude than I_Na_: when R_S_ is equal to 5 MΩ (in orange), alteration in the shape of the recordings is significant only when current amplitude is tenfold higher than Na^+^ currents (Fig. 5B, bottom center). However, a reduction in current amplitude is readily obtained for intermediate R_S_ and current amplitudes. When R_S_ reaches 15 MΩ and current amplitude is equal to several tens of nA, conditions routinely observed in automated patch-clamp with stable cell lines^7,8^, the model predicts a major modification of the activation curve and apparition of a delayed inactivation (Fig. 5B, bottom right). When R_S_ is not null, increasing peak current amplitude up to 100 nA leads to a major shift in voltage-dependence of activation as follows: for a peak current of 100 nA, R_S_ of 5 and 15 MΩ induces − 9 mV and − 16 mV shifts of the half-activation potential, respectively. The slope is also drastically increased by a factor of 1.8 and 2.4, respectively (Fig. 5C). Noteworthy, when Rs is 15 MΩ and amplitudes are in the order of several tens of nA, major voltage deviation occurs, decreasing the current amplitude by a factor of ten. This may falsely give the impression that the current is not high and thus that the introduced voltage error is negligible.

**Figure 6:**
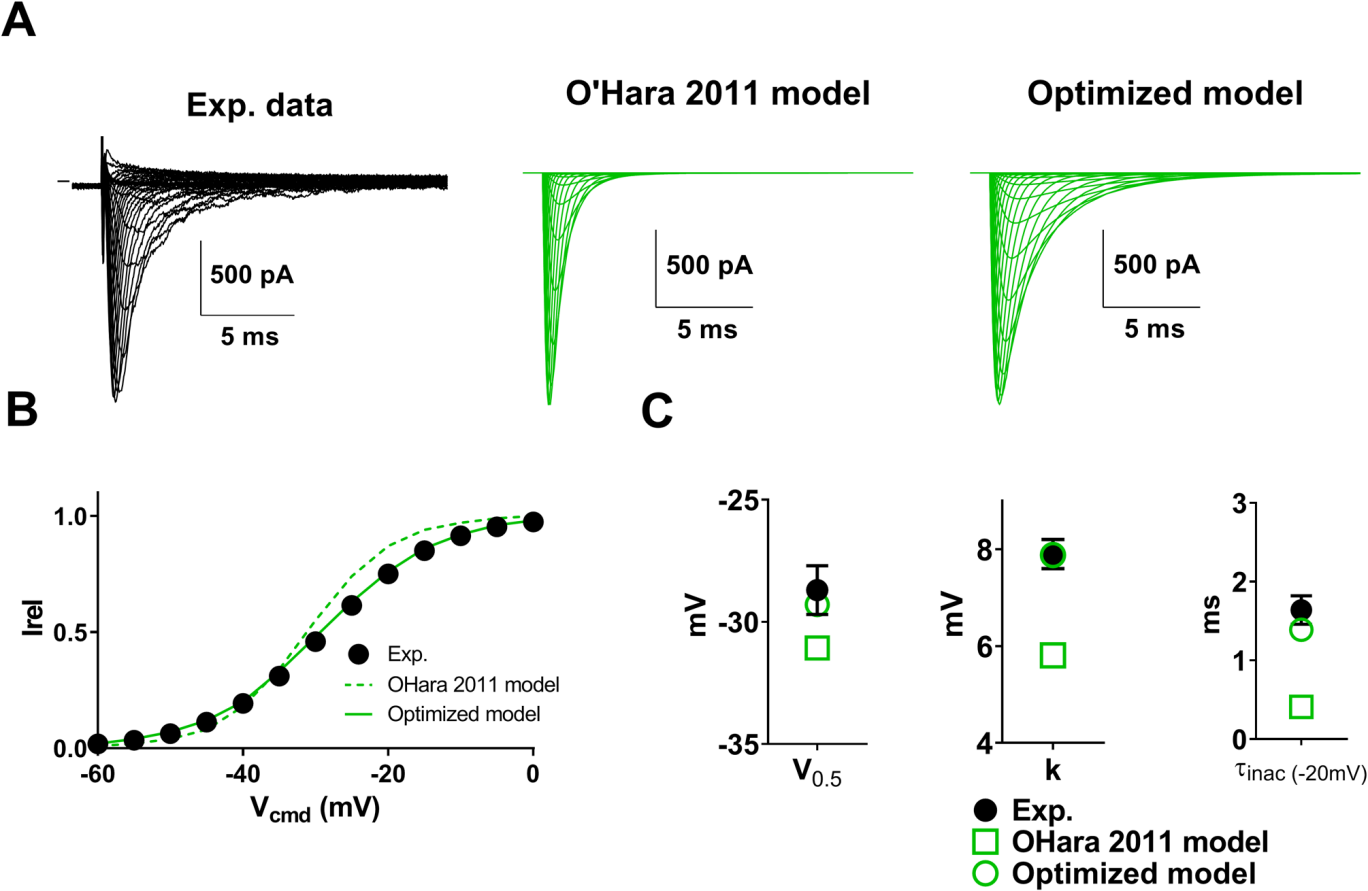
Optimization of the Kinetic model presented in Fig. 2 to fit the recordings of heterologously-expressed Na_v_1.5. **(A**) Left, representative, superimposed recordings of heterologously-expressed Na_v_1.5 during an activation voltage protocol (same as in Fig. 2). Middle, computed recordings using the same protocol, and the equation of^5^. Right, computed recordings using the same protocol, and the optimized model, to fit the biophysical characteristics of heterologously-expressed Na_v_1.5. (**B**) Activation curves in the three conditions presented in **(A)**. (**C**) Half-activation potentials, slopes and time constants of inactivation in the three conditions.

In order to test whether the model reproduces experimental data, we used a set of data of heterologously-expressed Na_v_1.5 currents recorded in COS-7 cells using manual voltage-clamp (qualitatively validated or not, to include highly ‘artefacted’, erroneous data). When using transient transfection systems, recorded currents are very variable from cell to cell, with peak currents measured at − 20 mV ranging from 391 pA to 17.8 nA in the chosen cell set (52 cells). We used this variability to study the effect of current amplitude on the activation curve properties. First, in order for the model to be as close as possible to experimental data, we modified the previously published Hodgkin-Huxley model to match the properties of the Na_v_1.5 current obtained in optimal experimental conditions (Fig. 6). We used as reference group, the cells presenting peak current amplitudes (measured at − 20 mV) in a range smaller than 1 nA (7 cells), and with R_S_ compensation allowing residual R_S_ of around 2 MΩ. The initial model (Fig. 3) suggests negligible alteration of V_0.5_ and k in these conditions. The model was then optimized by adjusting the Hodgkin-Huxley equations (Eqs. 9 and 10 in the “Methods” section) to obtain V_0.5_, k and inactivation time constants that are similar to averaged values of the 7 reference cells (Fig. 6B,C).

**Figure 7:**
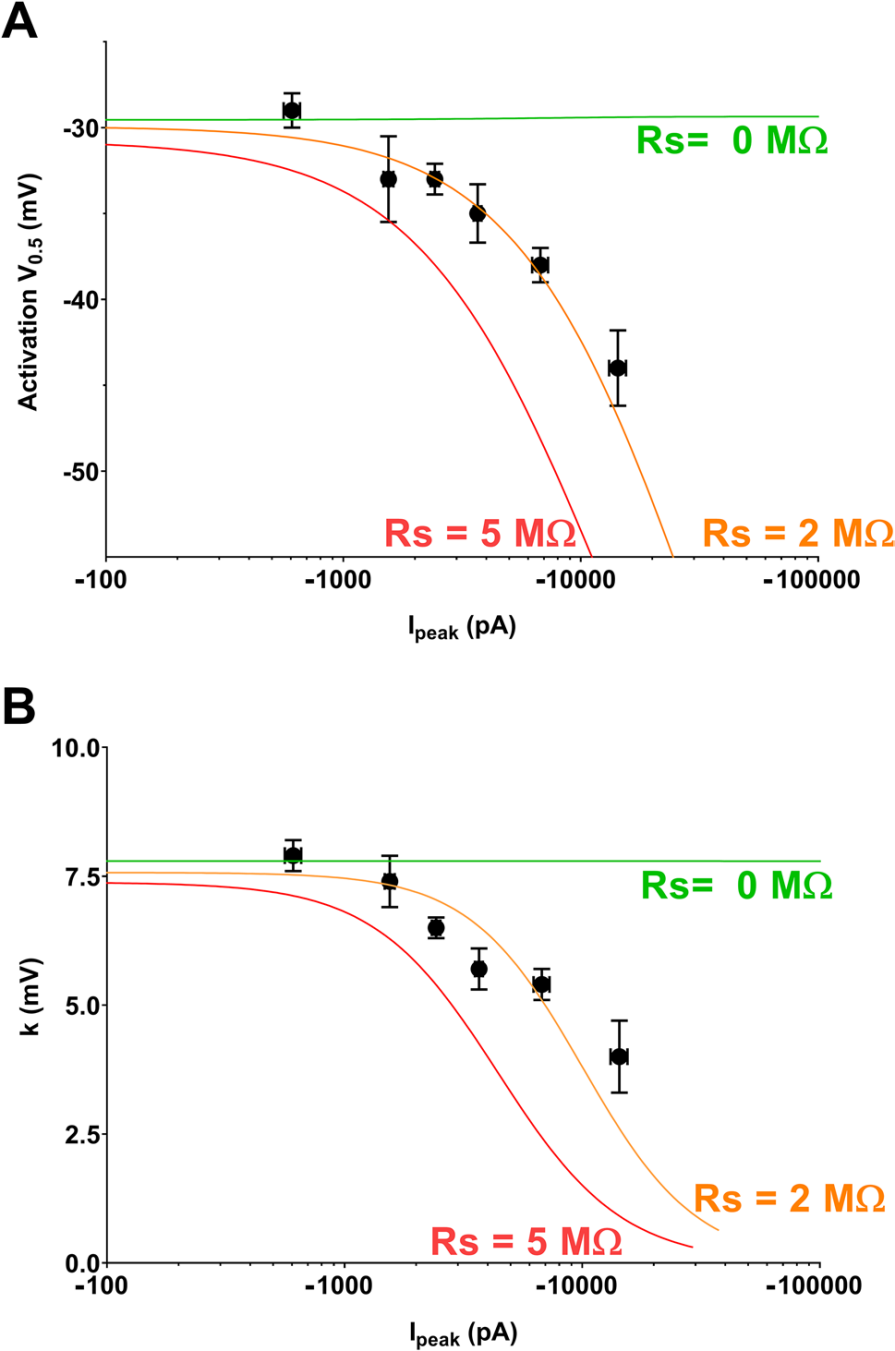
V_0.5_ vs. amplitude plots and slope *vs.* amplitude plots of heterologously-expressed Na_v_1.5 currents recorded in COS-7 using manual patch-clamp. (**A**) Symbols represent the mean ± sem of pooled experimental values of V_0.5_ as a function of mean ± sem values of current amplitudes. Lines correspond to fit of computed values obtained from the kinetic model that has been optimized in Fig. 6 and in which R_S_ has been set to 0, 2 and 5 M$\Omega$ (compensated C_m_ = 20 pF). (**B**) Pooled experimental values of the activation slope as a function of mean ± sem values of current amplitudes.

We then split the 52 cells in six groups according to current amplitude range (the 7 reference cells, then four groups of 10 cells, and a last group of 5 cells with a peak I_−20 mV_ greater that 10 nA), and plotted, for each group, the mean V_0.5_ (Fig. 7A) and k values (Fig. 7B) as a function of mean current amplitude. We observed a decrease in both V_0.5_ and k when current range increases. These relationships were successfully fitted by the computer model when R_S_ was set to 2 MΩ, which is close to the experimental value, after compensation (R_S_ = 2.3 ± 0.2 MΩ). In these conditions, if we accept maximal inward peak current amplitudes up to 7 nA, the error in V_0.5_ is below 10 mV and k remains greater than 5 mV. Experimentally, current amplitudes larger than 7 nA should be prevented or discarded, to prevent larger errors in evaluating V_0.5_ and k. Nevertheless, the benefit of such a representation (Fig. 7) is obvious as a correlation can be drawn between current amplitude and V_0.5_ and k values, with a more reliable evaluation of these values at low current amplitude levels.

**Figure 8:**
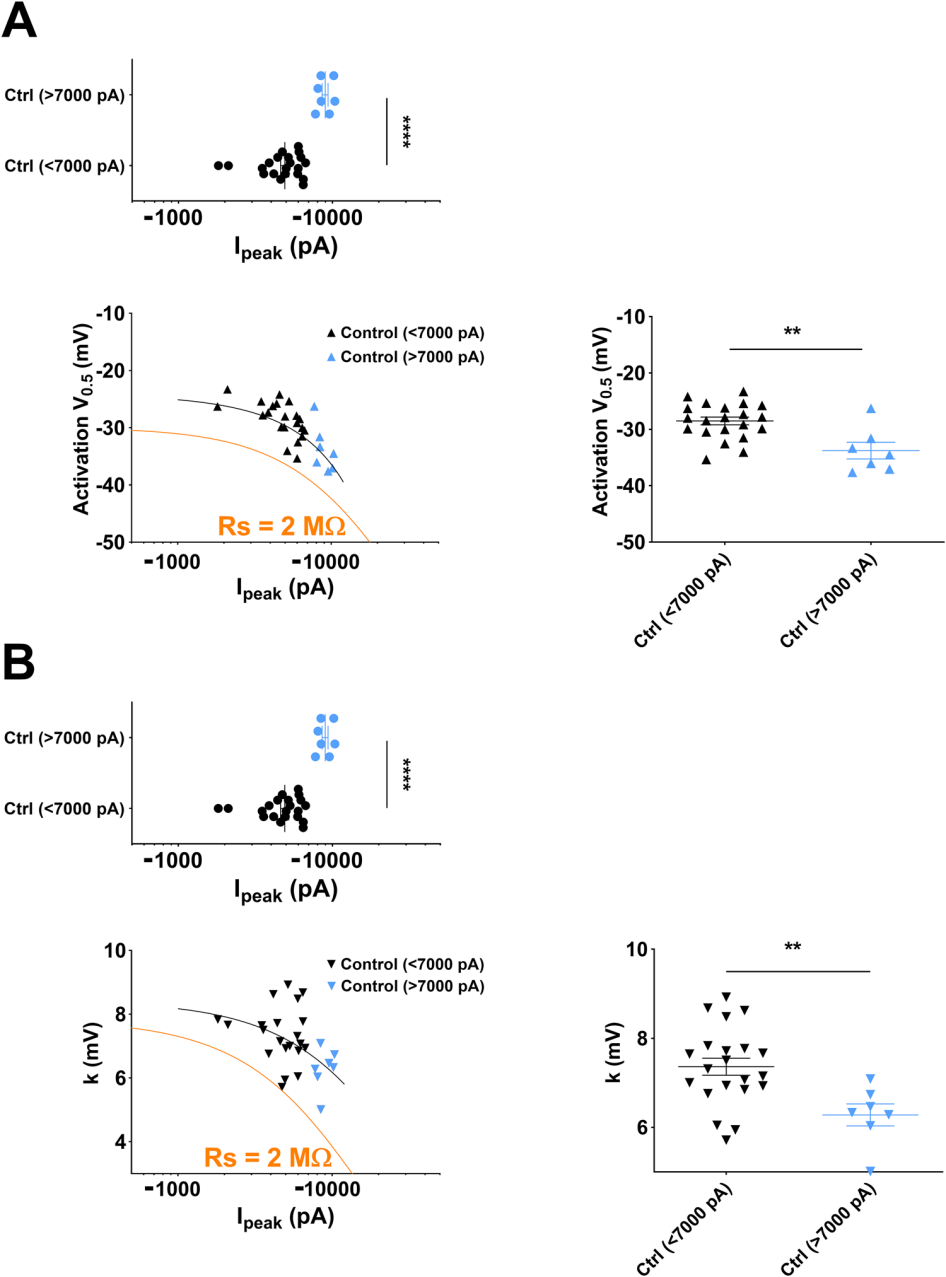
V_0.5_ vs. amplitude and slope *vs.* amplitude plots of I_Na_ measured from neonatal mouse ventricular cardiomyocytes using manual patch clamp. **(A**) Top, distribution of I_peak_ amplitudes of the data set. Blue data points correspond to a subset of cells with absolute amplitudes greater than 7000 pA. Left, symbols represent V_0.5_ values as a function of current amplitude values. The black line corresponds to exponential fit of the data, and the orange line corresponds to fit of computed values obtained from the kinetic model that has been optimized for heterologously-expressed Na_v_1.5 currents in COS-7 cells and in which R_S_ has been set to 2 M$\Omega$. Right, mean ± sem activation V_0.5_ for cells with current amplitudes smaller or greater than 7000 pA. ****, p < 0.0001, **, p < 0.01, Mann–Whitney test (I_peak_) and student’s t-test (V_0.5_). (**B**) Same as in A for the slope of the activation curve. ****, p < 0.0001, **, p < 0.01, Mann–Whitney test (I_peak_) and student’s t-test (k).

We then used a dataset of I_Na_ currents obtained from neonatal mouse ventricular cardiomyocytes, with values of current amplitudes that are frequently published, ranging from 1.8 to 10.3 nA, and using 80% series resistance compensation. We drew similar plots as in Fig. 7, of the experimental activation parameters, V_0.5_ and k, as a function of current amplitude in Fig. 8A,B, respectively, and we added the model of heterologously-expressed Na_v_1.5 currents (in orange) generated above. The model does not fit exactly to the data, suggesting that I_Na_ properties are slightly different in cardiomyocytes and transfected COS-7 cells. Interestingly, however, the exponential fits of the data follow the same trend, parallel to the COS-7 model, suggesting the same effect of Rs on V_0.5_ and k. Similar to transfected COS-7 cells, cardiomyocytes with currents greater than 7 nA display mean V_0.5_ ~ 5-mV more negative, and mean k ~ 1-mV smaller than cardiomyocytes with currents smaller than 7 nA (Fig. 8A,B), suggesting that using a 7 nA amplitude cut-off is appropriate. This comparison shows that differences in activation parameters may be blurred or exaggerated by inappropriate data pooling of cells with excessive current amplitude.

**Figure 9:**
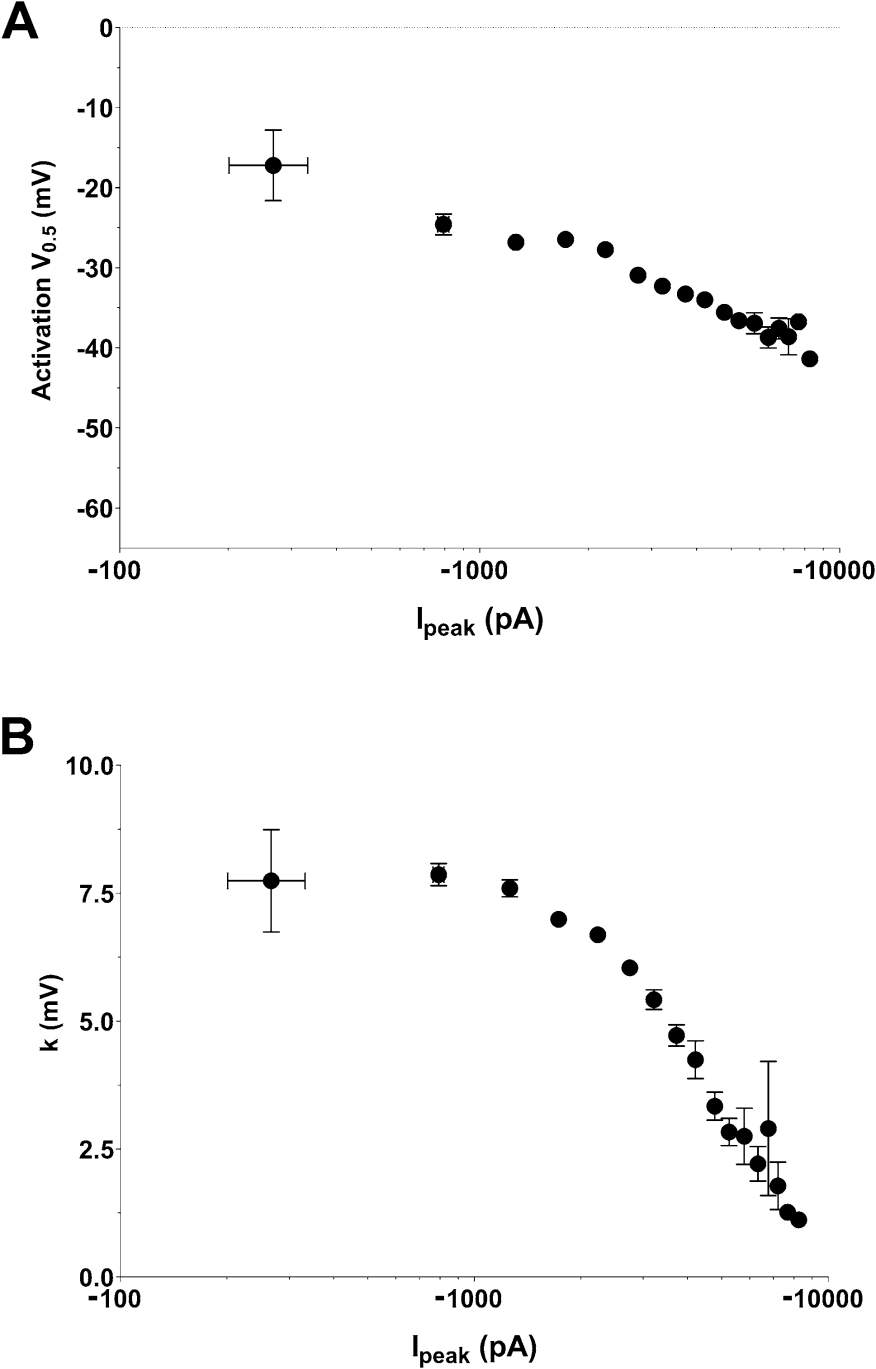
V_0.5_ vs. amplitude plots and slope *vs.* amplitude plots for I_Na_ in HEK293 cells stably expressing Na_v_1.5 measured using a Nanion Syncropatch 384PE. **(A**) Symbols represent the mean ± sem of pooled values of V_0.5_ as a function of mean ± sem values of current amplitudes. Data points were pooled by 500 pA amplitudes intervals (0–500, 500–1000, etc.) (**B**) Pooled experimental values of the activation slope as a function of mean ± sem values of current amplitudes.

Finally, we used a set of data from HEK293 cells stably expressing the Na_v_1.5 channels, obtained using the automated patch-clamp set-up Syncropatch 384PE (Fig. 9). Cells were grouped by intervals of 500 pA: 0–500 pA, 500–1000 pA, etc. The first experimental group has a mean inward current amplitude lower than the reference group of transfected COS-7 cells (− 267 ± 67 pA, n = 7 vs − 608 ± 48 pA, n = 7, respectively). It should be noticed that in this amplitude range, activation parameters are more difficult to determine. This is reflected by the large s.e.m. values for mean V_0.5_ and k. We postulate that HEK293 endogenous currents may non-specifically affect the properties of the recorded currents when they are in the 0–500 pA range. For the following groups with larger I_Na_ amplitudes, V_0.5_ seems to be stable. Hence, a V_0.5_ value around − 25 mV appears to be reliable. When current amplitudes are lower than 3.5 nA, the V_0.5_ change is less than 10 mV and k remains greater than 5 mV. Therefore, it is essential to perform experiments in conditions in which the inward current value is comprised between 500 pA and 3.5 nA when using such an automated patch-clamp system, and to exclude data with higher peak current amplitudes. These limits are more stringent than for manual patch-clamp as seen above (7 nA), but this is consistent with the limited compensation capabilities of some automated patch-clamp systems demonstrating slow response time for R_S_ compensation to avoid over-compensation and consequent current oscillation that can lead to seal disruption.

## Discussion

Even though effects of series resistance have been described very early^3^, a lot of published measured currents are in the range of several nA, which often leads to incorrect voltage clamp. We developed a simple model, using published kinetic models of ion currents, to simulate and describe such a caveat. We used both an inward current generated by a voltage-gated Na^+^ channel and an outward current generated by a voltage-gated K^+^ channel, both of them characterized by fast activation kinetics. Using these models and experimental recordings, we observed that large series resistance may give erroneous activation curves (Figs. 1,2,3,5,7,8,9) and dose–response curves (Fig. 4).

A similar mathematical model, taking into account the R_S_ impact has been used to study the causes of variability of current recordings obtained from the voltage-gated K^+^ channel K_v_11.1^9^. Here we used such a model to provide a guideline focusing on parameters that the manipulator can easily act on: current amplitude and R_S._

We observed that the activation parameters of cardiac voltage-gated Na^+^ current I_Na_ are much more sensitive than those of the cardiac voltage-gated K^+^ current I_to_: a current amplitude range of 10 nA combined with a R_S_ of 5 MΩ shows almost no alteration of the activation curve of the voltage-gated K^+^ channel (Fig. 5B, center and Fig. 5C, middle), whereas the same condition with the voltage-gated Na^+^ channel, shows a major alteration of the activation curve (Fig. 2B, bottom right and Fig. 3C, right). The simplest interpretation of this observation may be associated with the fact that, for Na^+^ channels, the increase in Na^+^ entry induced by depolarization further depolarizes the membrane and creates instability. For K^+^ channels, the increase of K^+^ outflow induced by depolarization tends to repolarize the membrane and limits instability. However, in extreme cases, repolarization prevents the occurrence of inactivation, leading to delayed inactivation (Fig. 5B, bottom right).

For the voltage-gated Na^+^ channel Na_v_1.5, we concluded that it is essential to prevent recording inward current amplitudes greater than 7 nA when residual R_S_ is around 2 MΩ to get a reasonable estimate of the activation gate characteristics in the manual patch-clamp technique (Fig. 7). When using an automated patch system, the limit is lowered to 3.5 nA (Fig. 9).

In order to test for activation changes, induced, for example, by drugs, mutations or post-translational modifications that are associated with current amplitude changes, it is advisable to generate plots of V_0.5_ or K as a function of I_peak_ in both conditions to early detect artefacts due to excessive current amplitude. This a priori caution will allow adapting experimental conditions to record currents below 7 nA.

To summarize, we suggest simple guidelines for the voltage-gated Na^+^ channel Na_v_1.5:

1. Always compensate R_S_ as much as possible,
2. R_S_ values around 2 MΩ after compensation allow recordings with a maximal inward current of 7 nA in manual patch-clamp,
3. Using Nanion Syncropatch 384PE, recordings with a maximal inward current of 3.5 nA can be used.

These guidelines may be extended to other Na_V_ isoforms contingent of generation of plots as in Figs. 7 and 9.

The guidelines are less stringent to record reliable K^+^ outward currents. However, one should always compensate R_S_ as much as possible. From Fig. 5B,C, for R_S_ values up to 5 MΩ after compensation, recordings with a maximal current of 10 nA will be highly reliable. With Nanion Syncropatch384PE, R_S_ values up to 15 MΩ after compensation allow recordings with a maximal current of 10 nA, but inhibitors or various transient transfection conditions should be used to make sure that the measured current amplitude is not saturating due to voltage deviation (Fig. 5B, bottom right).

For any current generated by voltage-gated channels, it is judicious to draw activation slope vs. amplitude plots and activation V_0.5_ vs. amplitude plots in a preliminary study to determine adapted conditions, a prerequisite to obtain reliable data and results.

For any other ion-channel type—ligand-gated, lipid-gated, regulated by second messengers or else—low membrane resistance i.e. high expression of active ion channels associated with high R_S_ values will also interfere with adequate voltage command and current measurements.

Several simple adaptations can be made to reach optimal experimental conditions:

1. R_S_ values are much lower when pipettes with low resistance (‘large’ pipettes) are used. When using amplifiers combining R_S_ and C_m_ compensation, suppression of pipette capacitance currents is of high interest since uncompensated pipette capacitance has a detrimental effect on the stability of the series resistance correction circuitry. This can be achieved by the use of borosilicate glass pipettes and wax or Sylgard coating^10^. When using Nanion Syncropatch384PE or other automated patch-clamp systems, low resistance chips are preferred.
2. When over-expressed channels are studied, transfection has to be adapted to produce a reasonable amount of channels to generate the desired current amplitude, or, when cell lines stably expressing the channel of interest are used, the clones generating the desired current amplitude range are preferably chosen. Any current, including native currents, can also be reduced when pipette and extracellular concentrations of the carried ion are reduced. In addition, the concentration gradient can be changed to limit the electrochemical gradient. Also inhibitors, such as TTX for Na_V_ channels, may be used at low concentration to reduce the current amplitude, as long as the inhibitor does not modify the biophysics of the WT and/or mutant channels, and it does not interfere with the action of other pharmacological compounds.

Therefore, any patch-clamp experiment needs to be carefully designed to reach appropriate conditions, guaranteeing rigorous analysis of the current. Finally, in native cells (excitable or non-excitable), the current passing through an ion channel type is always recorded in combination with other currents (leak current, at the minimum), and is generally isolated pharmacologically (e.g., TTX) or through other means, all involving subtraction of currents (e.g., P/n). The voltage error caused by R_s_ also depends on these other currents. Thus, it would be interesting to also model this situation to have a more integrated view of R_s_-induced incorrect voltage clamp.

## Methods

### Computer models

#### Application to whole-cell ion currents

I_Na_ and I_to_ currents were modeled using a Hodgkin–Huxley model of channel gating based on previously published models (O'Hara et al., 2011).

For cardiac I_Na_, we did not include the slow component of h, which only represents 1% of h inactivation (O'Hara et al., 2011).

1 $${m}_{\infty }=\frac{1}{1+\mathrm{exp}\left(-\frac{Vm+39.57}{9.871}\right)}$$

2 $${\tau }_{m}=\frac{1}{6.765\times \mathrm{exp}\left(\frac{Vm+11.64}{34.77}\right)+8.552\times \mathrm{exp}\left(-\frac{Vm+77.42}{5.955}\right)}$$

3 $${j}_{\infty }=\frac{1}{1+\mathrm{exp}\left(\frac{Vm+82.9}{6.086}\right)}$$

4 $${\tau }_{j}=2.038+\frac{1}{0.02136\times \mathrm{exp}\left(-\frac{Vm+100.6}{8.281}\right)+0.3052\times \mathrm{exp}\left(\frac{Vm+0.9941}{38.45}\right)}$$

5 $${h}_{\infty }=\frac{1}{1+\mathrm{exp}\left(\frac{Vm+82.9}{6.086}\right)}$$

6 $${\tau }_{h}=\frac{1}{1.432\times {10}^{-5}\times \mathrm{exp}\left(-\frac{Vm+1.196}{6.285}\right)+6.149 \times \mathrm{exp}\left(\frac{Vm+0.5096}{20.27}\right)}$$

The time-dependent gate values (m, h and j), were computed at every time step^11^ with an “adaptive time-step” method as:

7a $${y}_{t+tstep}={y}_{\infty }- \left({y}_{\infty -}{y}_{t}\right)\times\mathrm{exp}(-\mathrm{t}/\mathrm{tstep})$$

with y being the time-dependent gate value, and tstep, an adaptive time step. tstep was initialized to 0.1 µs, doubled when all the relative variations of m, h, or j were smaller than 0.5 × 10^–5^ and was halved when one of the relative variations of m, h, and j was greater than 10^–5^. When this limit was reached, the computation went one tstep backward and repeated again with the reduced tstep value to prevent divergence.

To validate this method, we also used an "LSODE" method (cf. example in supplemental Fig. 1). m, h and j were solved as:

7b $$\frac{dy}{dt}=\frac{{y}_{\infty } -y}{{\tau }_{y}}$$

using R software (v3.6.3, https://www.r-project.org) and the LSODE^12^ method from deSolve package (v1.28).

In the most critical condition: with a large Na^+^ current (Gmax = 6 µS) and large series resistance (Rs = 5 MΩ), both methods gave identical Na^+^ currents (cf. supplemental Fig. 1). Therefore, the “adaptive time-step” method was used to compute m, h and j values.

I_Na_ was calculated as follows:

8 $${I}_{Na}= {G}_{Na}\times \left(Vm- {E}_{Na}\right)\times {m}^{3}\times j \times h$$

with ${E}_{Na}= \frac{R T}{z F}\mathrm{log}\left(\frac{\left[Na^{+}\right]out}{\left[Na^{+}\right]int}\right)$, [Na^+^]*out* = 145 mM and [Na^+^]*in* = 10 mM.

To model overexpressed Na_v_1.5 currents, we adjusted some parameters, shown in bold, to fit the characteristics of the current when peak amplitude is less than 1 nA (cf results section and Fig. 6).

9 $${m}_{\infty }=\frac{1}{1+\mathrm{exp}\left(-\frac{Vm+\bf42.57}{\bf12}\right)}$$

10 $${\tau }_{h}=\frac{\bf4}{1.432 \times{10}^{-5}\times \mathrm{exp}\left(-\frac{Vm+1.196}{6.285}\right)+6.149 \times \mathrm{exp}\left(\frac{Vm+0.5096}{20.27}\right)}$$

To model cardiac I_to_, we did not include the CaMK dependent component, since at low Ca^2+^ pipette concentration (< 100 nM), this component is negligible (2%) (O'Hara et al., 2011).

11 $${a}_{\infty }=\frac{1}{1+\mathrm{exp}\left(-\frac{Vm-14.34}{14.82}\right)}$$

12 $${\tau }_{a}=\frac{1.0515}{\frac{1}{1.2089\times (1+\mathrm{exp}\left(-\frac{Vm-18.41}{29.38}\right))}+\frac{3.5}{1+\mathrm{exp}\left(\frac{Vm+100}{29.38}\right)}}$$

13 $${i }_{\infty }=\frac{1}{1+\mathrm{exp}\left(\frac{Vm+43.94}{5.711}\right)}$$

14 $${\tau }_{i,fast}=4.562+\frac{1}{0.3933\times \mathrm{exp}\left(-\frac{Vm+100}{100}\right)+0.08004 \times \mathrm{exp}\left(\frac{Vm+50}{16.59}\right)}$$

15 $${\tau }_{i,slow}=23.62+\frac{1}{0.001416\times \mathrm{exp}\left(-\frac{Vm+96.52}{59.05}\right)+1.78\times{10}^{-8}\times \mathrm{exp}\left(\frac{Vm+114.1}{8.079}\right)}$$

16 $${A}_{i,fast}=\frac{1}{1+\mathrm{exp}\left(\frac{Vm-213.6}{151.2}\right)}$$

17 $${A}_{i,slow}=1-{A}_{i,fast}$$

18 $$i={A}_{i,fast}\times {i}_{fast}+{A}_{i,slow}\times {i}_{slow}$$

a, ${i}_{fast}$ and ${i}_{slow}$ were computed at every time step^11^ using the “adaptive time-step” method (see above).

I_to_ was calculated as follows:

19 $${I}_{to}= {G}_{to}\times \left(Vm- {E}_{K}\right)\times a\times i$$

with ${E}_{K}= \frac{R T}{z F}\mathrm{log}\left(\frac{\left[K^{+}\right]out}{\left[K^{+}\right]int}\right)$, [K^+^]*out* = 5 mM and [K^+^]*in* = 145 mM.

For details on the kinetic models, please see^5^.

Membrane potential was computed as follows at each time step:

20 $$\frac{dVm}{dt}=\frac{Vcmd -Vm}{{R}_{s}\times {C}_{m}}- \frac{i}{{C}_{m}}$$

We hypothesized that the amplifier response time was not limiting. Membrane capacitance used was 20 pF and considered electronically compensated. Errors due to poor space clamp were considered negligible in small cells like COS-7 cells but it is worth mentioning that they should potentially be taken into account in bigger cells such as cardiomyocytes and in cells with complex morphologies such as neurons. Noteworthy, in some situations, specific protocols can reduce these artefacts linked to poor space-clamp^13^. Beyond technical issues due to the patch pipette, additional resistances, due to the narrow T-tubular lumen, are also not negligible in cardiac cell T-tubules and lead to delay in T-tubular membrane depolarization^14^.

#### Application to pharmacological investigations

Before investigating the effects of TTX, we computed the incidence of peak current amplitude at − 20 mV on its measured value with various R_s_ values (Fig. 4A). TTX effects were modeled by first constructing the theoretical dose–response curve with R_s_ = 0 MΩ. Knowing the experimental IC_50_ and Hill coefficient^6^, we calculated the TTX concentrations necessary to get 0.75 of the current (G_Na_ = 1.5 µS instead of G_Na_ = 2 µS in the absence of TTX), 0.5 (G_Na_ = 1 µS instead of G_Na_ = 2 µS in the absence of TTX), 0.3, … and 10^–3^ of the current. Then, for a given R_s_ (2 or 5 MΩ), we used the relationship between theoretical and observed measured values of peak current (Fig. 4A) to deduce the corresponding measured I_peak_ value of the residual current after TTX application. For instance, when R_s_ = 5 MΩ, for a theoretical current of − 27 nA, the measured current is about − 13 nA (see in Fig. 4A red chart). The effects of a 50% reduction of the theoretical value of − 27 nA (corresponding to the effect of 2.3 µM TTX, the IC_50_ value) results in a measured remaining current of − 8.3 nA when R_s_ = 5 MΩ (see in Fig. 4A red chart). Therefore, the apparent effect of 2.3 µM TTX on a measured current of − 13 nA is modeled by a − 8.3/− 13 ≈ 0.64 factor on G_Na_ in Eq. (8). Similar computations have been conducted for different control current amplitudes, Rs values, and TTX “doses”, and the corresponding dose–response curves have been built.

#### Cell culture and transfection

The African green monkey kidney-derived cell line, COS-7, was obtained from the American Type Culture Collection (CRL-1651) and cultured in Dulbecco’s modified Eagle’s medium (GIBCO) supplemented with 10% fetal calf serum and antibiotics (100 IU/mL penicillin and 100 µg/mL streptomycin) at 5% CO_2_ and 95% air, maintained at 37 °C in a humidified incubator. Cells were transfected in 35-mm Petri dishes when the culture reached 50–60% confluence, with DNA (2 µg total DNA) complexed with jetPEI (Polyplus transfection) according to the standard protocol recommended by the manufacturer. COS-7 cells were co-transfected with 200 ng of pCI-*SCN5A* (NM_000335.4), 200 ng of pRC-*SCN1B* (NM_001037) (kind gifts of AL George, Northwestern University, Feinberg School of Medicine) and 1.6 µg pEGFP-N3 plasmid (Clontech). Cells were re-plated onto 35-mm Petri dishes the day after transfection for patch-clamp experiments. HEK293 cells stably expressing hNa_v_1.5 were cultured in Dulbecco’s Modified Eagle’s Medium (DMEM) supplemented with 10% fetal calf serum, 1 mM pyruvic acid, 2 mM glutamine, 400 µg/ml of G418 (Sigma), 100 U/mL penicillin and 100 μg/mL streptomycin (Gibco, Grand Island, NY) at 5% CO_2_ and 95% air, maintained at 37 °C in a humidified incubator.

#### Statement on the use of mice

All investigations conformed to directive 2010/63/EU of the European Parliament, to the Guide for the Care and Use of Laboratory Animals published by the US National Institutes of Health (NIH Publication No. 85-23, revised 1985) and to local institutional guidelines.

#### Neonatal mouse ventricular cardiomyocyte isolation and culture

Single cardiomyocytes were isolated from the ventricles of mouse neonates aged from postnatal day 0 to 3 by enzymatic and mechanical dissociation in a semi-automated procedure by using the Neonatal Heart Dissociation Kit and the GentleMACS™ Dissociator (Miltenyi Biotec). Briefly, hearts were harvested, and the ventricles were separated from the atria, and digested in the GentleMACS™ Dissociator. After termination of the program, the digestion was stopped by adding medium containing Dulbecco’s Modified Eagle’s Medium (DMEM) supplemented with 10% horse serum, 5% fetal bovine serum and 100 U/ml penicillin and 100 μg/ml streptomycin. The cell suspension was filtered to remove undissociated tissue fragments, and centrifugated. The cell pellet was resuspended in culture medium, and the cells were plated in 60 mm-diameter Petri dishes at 37 °C for 1.5 h. The non-plated myocytes were then resuspended, plated on laminin-coated dishes at a density of 50 000 cells per plate, and incubated in 37 °C, 5% CO_2_: 95% air incubator. After 24 h-plating, medium was replaced by DMEM supplemented with 1% fetal bovine serum and 100 U/mL penicillin and 100 μg/mL streptomycin, and electrophysiological experiments were performed 48 h following isolation.

#### Manual electrophysiology on transfected COS-7 cells

One or 2 days after splitting, COS-7 cells were mounted on the stage of an inverted microscope and constantly perfused by a Tyrode solution maintained at 22.0 ± 2.0 °C at a rate of 1–3 mL/min; HEPES-buffered Tyrode solution contained (in mmol/L): NaCl 145, KCl 4, MgCl_2_ 1, CaCl_2_ 1, HEPES 5, glucose 5, pH adjusted to 7.4 with NaOH. During Na^+^ current recording, the studied cell was locally superfused^15^ with a extracellular solution used to prevent endogenous K^+^ currents, containing (in mmol/L): NaCl, 145; CsCl, 4; CaCl_2_, 1; MgCl_2_, 1; HEPES, 5; glucose, 5; pH adjusted to 7.4 with NaOH. Patch pipettes (tip resistance: 0.8 to 1.3 MΩ) were pulled from soda lime glass capillaries (Kimble-Chase) and coated with dental wax to decrease pipette capacitive currents. The pipette was filled with Na^+^ intracellular medium containing (in mmol/L): CsCl, 80; gluconic acid, 45; NaCl, 10; MgCl_2_, 1; CaCl_2_, 2.5; EGTA, 5; HEPES, 10; pH adjusted to 7.2 with CsOH. Stimulation and data recording were performed with pClamp 10, an A/D converter (Digidata 1440A) and an Axopatch 200B (all Molecular Devices) or an Alembic amplifier (Alembic Instruments). Currents were acquired in the whole-cell configuration, filtered at 10 kHz and recorded at a sampling rate of 33 kHz. Before series resistance compensation, a series of 50 25-ms steps were applied from − 70 mV to − 80 mV to subsequently calculate off-line C_m_ and R_S_ values from the recorded current. To generate the Na_v_1.5 activation curve, the membrane was depolarized from a holding potential of − 100 mV to values between − 80 mV and + 50 mV (+ 5-mV increment) for 50 ms, every 2 s. Activation curves were fitted by a Boltzmann equation: G = G_max_/(1 + exp (− (V_m_ − V_0.5_)/k)), in which G is the conductance, V_0.5_ is the membrane potential of half-activation and k is the slope factor. For Fig. 7, cells were grouped by 10, except the first group which includes the cells with a absolute peak I_− 20 mV_ of less that 1000 pA (n = 7) and the last group which includes the cells with a peak I_− 20 mV_ greater that 10 nA (n = 5).

#### Electrophysiology on cardiomyocytes

Whole-cell Na_v_ currents were recorded at room temperature 48 h after cell isolation with pClamp 10, an A/D converter (Digidata 1440A) and an Axopatch 200B amplifier (all Molecular Devices). Current signals were filtered at 10 kHz prior to digitization at 50 kHz and storage. Patch-clamp pipettes were fabricated from borosilicate glass (OD: 1.5 mm, ID: 0.86 mm, Sutter Instrument, Novato, CA) using a P-97 micropipette puller (Sutter Instrument), coated with wax, and fire-polished to a resistance between 0.8 and 1.5 MΩ when filled with internal solution. The internal solution contained (in mM): NaCl 5, CsF 115, CsCl 20, HEPES 10, EGTA 10 (pH 7.35 with CsOH, ~ 300 mosM). The external solution contained (in mM): NaCl 20, CsCl 103, TEA-Cl (tetraethylammonium chloride) 25, HEPES 10, glucose 5, CaCl_2_ 1, MgCl_2_ 2 (pH 7.4 with HCl, ~ 300 mosM). All chemicals were purchased from Sigma. After establishing the whole-cell configuration, stabilization of voltage-dependence of activation and inactivation properties was allowed during 10 min. Before series resistance compensation, series of 25-ms steps were applied from − 70 mV to − 80 mV and to − 60 mV to subsequently off-line calculate C_m_ and R_S_ values from the recorded currents. After compensation of series resistance (80%), the membrane was held at a HP of − 120 mV, and the voltage-clamp protocol was carried out as follows. To determine peak Na^+^ current–voltage relationships, currents were elicited by 50-ms depolarizing pulses to potentials ranging from − 80 to + 40 mV (presented at 5-s intervals in 5-mV increments) from a HP of − 120 mV. Peak current amplitudes were defined as the maximal currents evoked at each voltage, and were subsequently leak-corrected. To analyze voltage-dependence of activation properties, conductances (G) were calculated, and conductance-voltage relationships were fitted with a Boltzmann equation. Data were compiled and analyzed using ClampFit 10 (Axon Instruments), Microsoft Excel, and Prism (GraphPad Software, San Diego, CA).

#### High-throughput electrophysiology

Automated patch-clamp recordings were performed using the SyncroPatch 384PE from Nanion (München, Germany). Single-hole, 384-well recording chips with medium resistance (4.77 ± 0.01 MΩ, n = 384) were used for recordings of HEK293 cells stably expressing human Na_v_1.5 channel (300 000 cells/mL) in whole-cell configuration. Pulse generation and data collection were performed with the PatchControl384 v1.5.2 software (Nanion) and the Biomek v1.0 interface (Beckman Coulter). Whole-cell recordings were conducted according to the recommended procedures of Nanion. Cells were stored in a cell hotel reservoir at 10 °C with shaking speed at 60 RPM. After initiating the experiment, cell catching, sealing, whole-cell formation, buffer exchanges, recording, and data acquisition were all performed sequentially and automatically. The intracellular solution contained (in mM): 10 CsCl, 110 CsF, 10 NaCl, 10 EGTA and 10 HEPES (pH 7.2, osmolarity 280 mOsm), and the extracellular solution contained (in mM): 60 NaCl, 4 KCl, 100 NMDG, 2 CaCl_2_, 1 MgCl_2_, 5 glucose and 10 HEPES (pH 7.4, osmolarity 298 mOsm). Whole-cell experiments were performed at a holding potential of − 100 mV at room temperature (18–22 °C). Currents were sampled at 20 kHz. Activation curves were built by 50 ms-lasting depolarization from − 80 mV to 70 mV (+ 5 mV increment), every 5 s. Activation curves were fitted by Boltzmann equation. Stringent criteria were used to include individual cell recordings for data analysis (seal resistance > 0.5 GΩ and estimated series resistance < 10 MΩ).

## Supplementary Information

Supplementary Information.
