# Supplementary material for: Computer modeling of whole-cell voltage-clamp analyses to delineate guidelines for good practice of manual and automated patch-clamp
Source: Sci Rep. 2021 Feb 8;11:3282. doi: 10.1038/s41598-021-82077-8 (PMC7870888; doi:10.1038/s41598-021-82077-8)
Supplement: Supplementary file 1 — Supplementary Information. [file 41598_2021_82077_MOESM1_ESM.pdf]

Computer modeling of whole-cell voltage-clamp analyses to delineate guidelines for good practice of manual and automated patch-clamp

Jérôme Montnach[1$], Maxime Lorenzini[1$], Adrien Lesage[1], Isabelle Simon[1], Sébastien Nicolas[1], Eléonore Moreau[1#], Céline Marionneau[1], Isabelle Baró[1], Michel De Waard[1,2], Gildas Loussouarn[1,*]

1. *Université de Nantes, CNRS, INSERM, l'institut du thorax, F-44000 Nantes, France.*
2. *LabEx « Ion Channels, Science & Therapeutics », Valbonne F-06560, France.*

[$] ***These authors contributed equally to this work.***

[#] Present address: Laboratoire Signalisation Fonctionnelle des Canaux Ioniques et des Récepteurs (SiFCIR), UPRES EA 2647, USC INRA 1330, SFR QUASAV 4207, UFR Sciences, Université d'Angers

*** Address correspondence to:**
Dr. Gildas Loussouarn
L'institut du thorax
INSERM UMR 1087 / CNRS UMR 6291
IRS-UN, 8 Quai Moncousu BP 70721
44007 Nantes cedex 1, France
Tel: +33 (0)2 2808 0150
E-mail: gildas.loussouarn@inserm.fr

Keywords: Voltage-gated ion channels – whole-cell voltage-clamp analyses – computer modeling - guidelines.

Relationship between ion current and the quality of voltage-clamp summarized in a limited number of equations.

For a given ionic species, the current *I* depends on the driving force applied to the carried ion to cross the channel. For Na^+ current:

$$I_{Na} = \frac{(V_m - E_{Na})}{R_m} \qquad \text{eq. s1}$$

with V_m the voltage difference across the cell membrane; R_m, the membrane resistance for Na^+ (inversely proportional to the number of Na^+ channels in the open state); E_{Na}, the reversal potential determined by the Nernst equation:

$$E_{Na} = \frac{RT}{zF} \ln([Na^+]_{out}/[Na^+]_{in}) \qquad \text{eq. s2}$$

where R is the universal gas constant (8.314 Joules per Kelvin per mole); *T*, the temperature in Kelvin; *z*, the valence of the ionic species (+1 for Na^+); *F*, the Faraday constant (96485 Coulombs per mole). $[Na^+]_{out}$ is the concentration of Na^+ in the extracellular solution and $[Na^+]_{in}$ in the intracellular medium (pipette solution).

The series resistance is a sum of resistances dependent on the pipette tip size and access into the cell. According to the Ohm law: $V = R \times I$

For R_S and R_m, two resistances in series:

$V_{cmd} = V_S + V_m$ and, from eq. s1, $V_m = R_m \times I_{Na} + E_{Na}$

$$V_{cmd} = (R_S \times I_{Na}) + (R_m \times I_{Na}) + E_{Na}$$

If most of the channels are closed or lowly expressed, $R_m \gg R_S$ and V_S is negligible when compared to V_m. In this case, $V_m \equiv V_{cmd}$.

When channels are opening, R_m decreases and *I* increases. As long as R_S remains low by using pipettes with large tip, and R_m remains several log higher than R_S (by maintaining a low channel expression), V_m remains fairly close to V_{cmd}. But if the channels are highly expressed, R_m can reach values in the range of R_S. In these conditions, V_S cannot be neglected when compared to V_m and V_m becomes a fraction of V_{cmd}, only.

For native Na^+ channels in cardiomyocytes, for example, the current amplitude can be lowered by reducing E_{Na} (eq. s2) *i.e.* by decreasing the concentration gradient, and by reducing the number of ions crossing the channels by partly substituting them with an equivalent non-permeant ion in the solutions (here Cs^+ for Na^+).

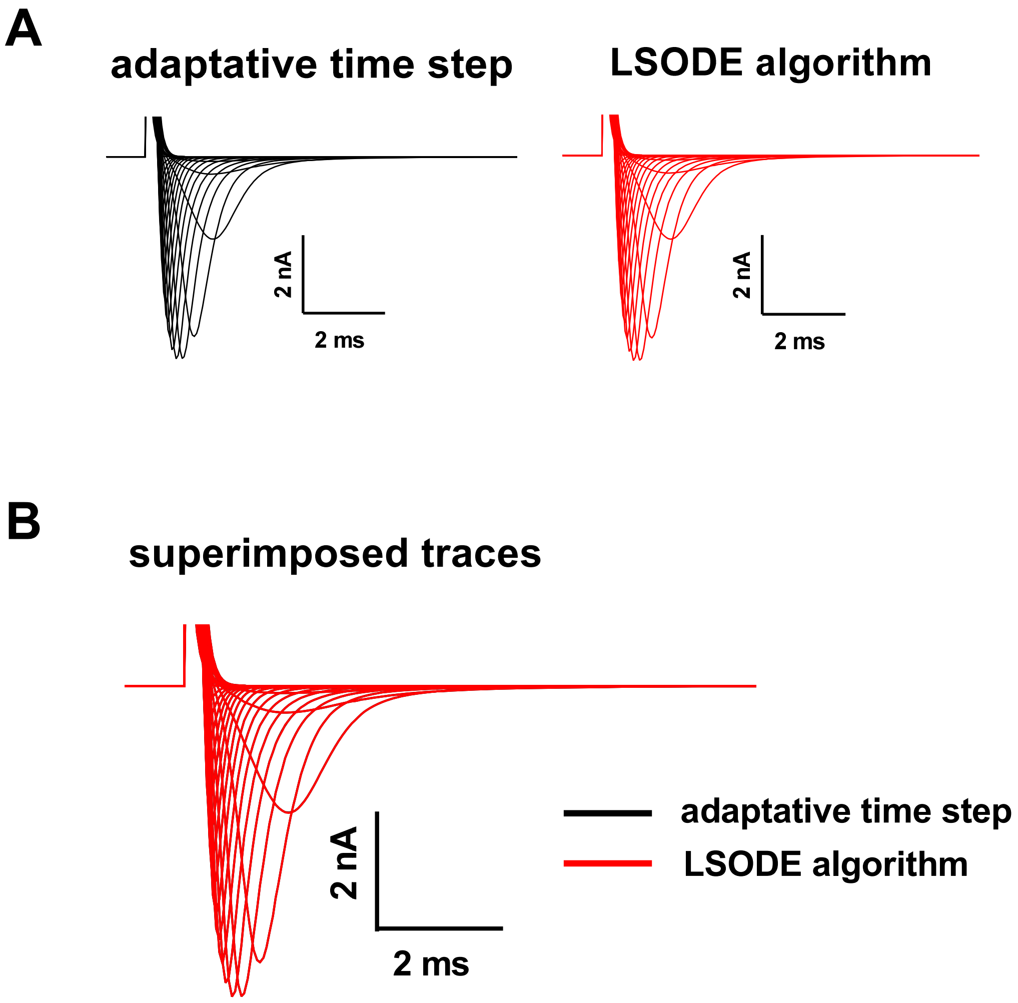

Supplemental figure 1: Comparison of the modeled I_{Na} current, with G_{max} = 6 µS and R_S = 5 MΩ (Figure 2B) using the two computation methods presented in the methods section.
